# Experiences using an instrument for non-technical skills in nurse anaesthesia education: a focus group study

**DOI:** 10.1186/s12909-022-03322-w

**Published:** 2022-04-04

**Authors:** Fiona M. Flynn, Berit T. Valeberg, Pia C. Bing-Jonsson, Anne M. Lyberg, Siri Tønnessen

**Affiliations:** 1 University of South-Eastern Norway, Postbox 235, 3603 Kongsberg, Norway; 2 OsloMet University and University of South-Eastern Norway, Kongsberg, Norway

**Keywords:** Nurse anaesthetist/anesthetist, Non-technical skills, Clinical supervision, Education, Clinical excellence, NANTS-no

## Abstract

**Background:**

Although there is an increasing amount of research on the use of structured behavioural assessment instruments for non-technical skills in a simulation or clinical setting, there is currently little research into how healthcare professionals experience using these instruments. The structured behavioural assessment instrument, Nurse Anaesthetists’ Non-Technical Skills-Norway, has recently been introduced to nurse anaesthesia education as a means of developing and assessing non-technical skills in clinical practice. The aim of this study was therefore to explore the experiences of Norwegian student nurse anaesthetists, their mentors and clinical supervisors on using the instrument in clinical practice.

**Methods:**

This study has a qualitative descriptive design. Data was collected through semi-structured interviews with four focus groups comprising twelve student nurse anaesthetists and thirteen mentors and clinical supervisors. The interviews were recorded and then transcribed verbatim. Data was analyzed using qualitative content analysis and an inductive approach.

**Results:**

Six categories were identified that represented the manifest content. One main theme: Forging a path towards clinical excellence was identified representing the latent content, and three themes that described the participants’ experiences with using the instrument:

Promotion of excellent non-technical skills: Raising awareness of non-technical skills ensured professional suitability and shaping of a professional identity; internalizing the skills could lead to changes in behaviour.

Promotion of cooperative learning: Mentoring was more structured, based on a common language and understanding and clearly defined roles; measurable progress enabled a more reliable and objective evaluation.

Promotion of organizational acceptance: A lack of familiarity with the instrument, and challenges with scoring and the terminology impeded acceptance.

**Conclusion:**

Increased awareness of non-technical skills when using Nurse Anaesthetists’ Non-Technical Skills-Norway contributes to a professionalization of the nurse anaesthetist role and mentoring/learning process in nurse anaesthesia education. Using Nurse Anaesthetists’ Non-Technical Skills-Norway promotes the ideal of clinical excellence, not only as an assessment instrument but also by guiding the student’s learning process. Despite a high level of commitment to using the instrument there is a need to promote further acceptance in the anaesthetic departments.

## Background

Structured behavioural assessment instruments for systematically developing and assessing non-technical skills as a means of enhancing performance and improving patient safety, are still relatively new and used in varying degrees by healthcare professionals [1]. By focusing on human factors, their purpose is to improve individual or team non-technical skills to reduce the risk of errors and adverse events that threaten patient safety [1, 2]. Non-technical skills are described as “cognitive, social and personal resource skills that complement technical skill, and contribute to safe and efficient task performance” [3]. They are regarded as the key to consistently high performance in the best practitioners and are an essential part of being professionally competent [3, 4]. There is increasing interest in the development and assessment of non-technical skills in anaesthesia internationally, but they are currently not systematically incorporated in the nurse anaesthesia curriculum in Norway.

Nurse anaesthesia education, scope of practice and potential for continuing professional development varies widely internationally [5–7]. In Norway, nurse anaesthetists are registered nurses, who have completed either a two-year masters’ degree or an eighteen-month post-registration program in nurse anaesthesia. This qualifies them among other things to independently induce and maintain general anaesthesia in patients classified as American Society of Anesthesiologists (ASA) class I or II [6–8]. Nurse anaesthetists work as part of a multidisciplinary surgical team, collaborating closely with anaesthesiologists in particular [8]. During anaesthesia, the nurse anaesthetist is responsible for maintaining homeostasis in the patient, often in acute situations that are both complex and dynamic. This requires high standards of competency and safety as advocated by the International Federation of Nurse Anesthetists (IFNA) [9]. It also demands a level of professionalism that encompasses recognizing and accepting responsibility for maintaining high levels of knowledge, skills, and professional values as well as an active commitment to self-appraisal and continuous professional development [5, 9].

Student nurse anaesthetists (SNAs) learn in clinical practice under the guidance of one or two designated mentors, who are experienced postgraduate nurse anaesthetists that act as role models, facilitating learning by sharing their craft and providing affirmative and formative feedback [10]. A mentor’s role involves guiding the SNAs through complex, dynamic and critical situations, while simultaneously ensuring that the patient receives optimal standards of anaesthesia care. The mentor is also responsible for carrying out assessments together with a clinical supervisor to ensure that the SNA has acquired the necessary skills [11]. Clinical supervisors act as a bridge between the educational and healthcare institutions. Their responsibilities include overseeing and organizing clinical practice for all the SNAs at a healthcare trust, teaching and supporting the SNAs and their mentors. Since clinical practice comprises a major part of the training, clinical supervision is an integral part of nurse anaesthesia education. Although some countries have national standards for mentoring, these do not currently exist in Norway, and a large number of nurse anaesthetists involved in clinical supervision lack formal training [12, 13].

The relationship between student and mentor plays an important role in both facilitating the SNA’s learning and strengthening professionalism [13, 14]. In order for SNAs to flourish, they need to be treated as equal partners in a relationship where dialogue and critical reflection can enhance learning [13, 15]. This kind of cooperative learning can be defined as “a set of processes which help people interact together in order to accomplish a specific goal or develop an end product which is usually content specific” [16]. While collaborative learning is often used to describe peer or group learning that occurs through social interaction, observation of more knowledgeable others and scaffolding [17], cooperative learning is more closely directed by a teacher or mentor [18]. However, cooperative and collaborative learning share several common assumptions regarding active learning. These include the mentor acting more as a facilitator, learning that is based on the mentor’s and student’s shared experiences, students taking responsibility for their learning and discussion that aids critical reflection [18].

Traditionally, SNAs’ learning in clinical practice has focused on specialized technical skills, however in recent years there has been increasing international awareness of the importance of non-technical skills in providing safe anaesthesia [2]. Facilitating a systematic development of non-technical skills in SNAs is dependent on several factors. The right conditions for learning must be created; a highly challenging but safe environment that stimulates active rather than passive learning [15]. There is also a need for a standardized conceptual model with a common taxonomy for observing, discussing and assessing clinical competencies in SNAs in order to provide feedback on areas that need addressing [19]. This kind of formative feedback is an integral part of the teaching/learning process and contributes to changing behaviour and developing expert skills [20].

**Fig. 1:**
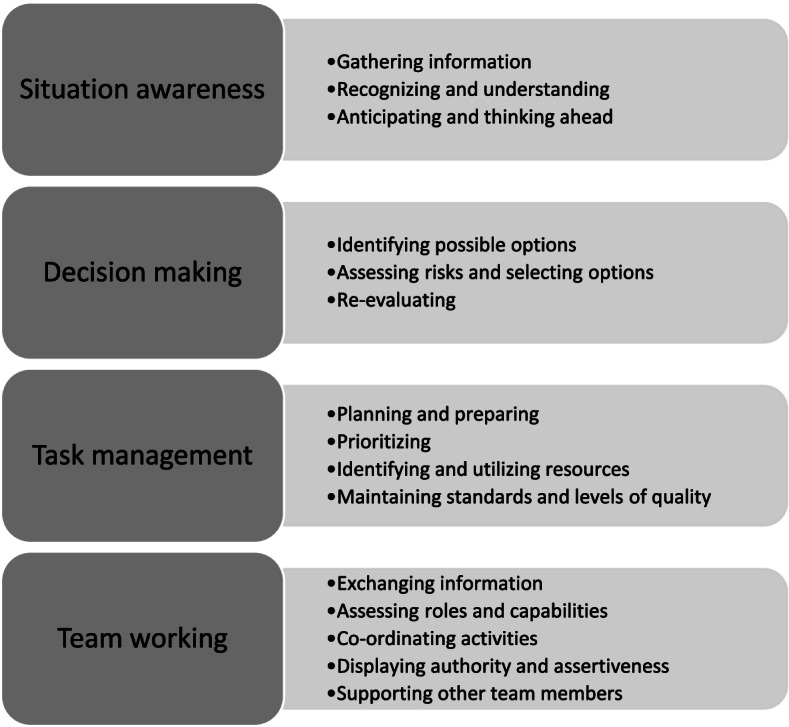
The NANTS-no structured behavioural assessment instrument

Several educational and healthcare institutions in Norway have adopted the Nurse Anaesthetists’ Non-Technical Skills – Norway (NANTS-no) structured behavioural assessment instrument as a means of providing formative feedback, encouraging critical reflection, and developing and assessing SNAs’ non-technical skills. NANTS-no is a behavioural marker system that has a hierarchical structure with four categories and fifteen elements (Fig. 1). Each element has behavioural markers with examples of good and poor behaviour. The instrument also has a 5-point behavioural rating scale (1–5) for non-technical skills, where 1 corresponds to behaviour that puts the patient’s life at risk and 5 corresponds to excellent behaviour.

NANTS-no was adapted from Anaesthetists Non-Technical Skills (ANTS) in 2014 to reflect the setting in which nurse anaesthetists work in Norway [21] and was originally intended for use by qualified nurse anaesthetists in professional development. However, it has been also tested for use in nurse anaesthesia education in Norway and demonstrated a high level of reliability (ICC = 0.8, Cronbach’s alpha > 0.9) and dependability (G coefficient = 0.83) [22, 23]. In addition to ANTS, there are similar instruments for other professions, such as anaesthetic practitioners, surgeons and scrub nurses [1].

NANTS-no has been used in clinical practice over the past few years at the university where this study was held, by both the SNAs and those involved in clinical supervision at the healthcare trusts. It is intended to be used as a means of self-assessment by the students and in evaluations by the mentors and clinical supervisors, where non-technical skills are rated according to the skills expected of a qualified nurse anaesthetist. Moreover, it has a formative purpose to aid feedback in clinical supervision. As part of their education, the SNAs are taught about the importance of non-technical skills and the use of NANTS-no prior to clinical practice.

Although there are a growing number of studies where non-technical skills are assessed in a simulation or clinical setting [1], there is currently little research describing how healthcare professionals experience using structured behavioural assessment instruments in clinical settings [24]. NANTS-no was tested for reliability prior to use in clinical supervision [23], and then used to demonstrate the development and assessment of SNAs’ non-technical skills during nurse anaesthesia education [11]. It was therefore considered important to gain an understanding of how mentors, clinical supervisors and SNAs experienced using the instrument. Thus, the purpose of this study was to explore both the instrument’s usefulness and usability in a new area, that of nurse anaesthesia education. Since there is a little research into users’ experiences with instruments for non-technical skills, this study may also provide useful information for other fields.

### Aim

The aim of the study was to explore the experiences of Norwegian student nurse anaesthetists, their mentors, and clinical supervisors with using NANTS-no in clinical practice during nurse anaesthesia education.

## Methods

### Design and setting

This study has a descriptive qualitative design [25]. Data was collected through semi-structured interviews with four focus groups [26] and analyzed using qualitative content analysis [27]. Focus group methodology was chosen as a means of gaining rich insight into the participants’ experiences using the instrument [28, 29]. It was assumed that interaction within the focus groups would provide richer data than individual interviews, by creating a synergy where shared and contradictory viewpoints would lead to a new and deeper understanding of the research subject [26].

The study was conducted at a Norwegian University, which has a two-year masters’ program in nurse anaesthesia (120 ECTS). Clinical practice comprises 45 ECTS of the course, and the University has clinical placements at a number of healthcare trusts in Norway. SNAs are assigned to an anaesthetic department in one of the healthcare trusts for clinical practice and mentored by qualified nurse anaesthetists with experience in the field. The majority of mentors and clinical supervisors attended a six-hour training session prior to the SNAs starting their clinical practice [30].

### Participants

A purposeful sampling strategy was used for recruiting participants that had experienced using NANTS-no and ensure maximum variation in the sample [26, 31]. SNAs from two different student cohorts as well as nurse anaesthetists involved in mentoring and clinical supervision of the students were invited to take part in the study. To ensure heterogeneity in the sample and different experiences, the SNAs were recruited at different stages in their education [26]. One cohort had just completed their first period of clinical practice while the other had completed all their clinical training. Purposeful sampling of the nurse anaesthetists involved in clinical supervision ensured variation in workplace, sex, and level of experience [31].

All participants were contacted directly via an email containing information about the study. Twelve SNAs agreed to participate, six from each cohort with an equal number of male and female participants in each group. The SNAs had been on clinical placement at seven different healthcare trusts. Thirteen nurse anaesthetists involved in clinical supervision also agreed to participate, representing five healthcare trusts. Several of those contacted showed interest in the study but were unable to attend owing to staffing and other difficulties. The thirteen participants included a mixture of mentors and clinical supervisors and formed two focus groups with eight participants in one group and five in the other. There was only one male in each group, and the participants had a range of two to thirty years’ experience as a nurse anaesthetist.

Sample size was guided by an appraisal of the study’s information power [32]. Although Malterud et al. state that their concept for guiding sample size is more ambiguous for focus group interviews, a critical appraisal of the study’s aim, sample specificity, use of theory, quality of interview dialogue and analysis strategy to determine information power was seen as a means of strengthening validity. Based on a provisional assessment of these criteria for determining information power and achieving data saturation, four focus groups were presumed adequate [26, 33]. This assessment was confirmed based on the dialogue quality in the interviews, which provided the multiple viewpoints and rich variations in data necessary for content analysis [33, 34]. Since the last focus group provided no new data, saturation was assumed.

### Data collection

Data was collected over a period of eighteen months between April 2019 and September 2020. This was in order to gain access to a new cohort of students and a richer data. All the interviews were held in one of the meeting-rooms at the University to avoid interruptions, with the first author acting as moderator. Two of the co-authors took the role of assistant moderator, S.T for three of the interviews and P.B-J for the fourth. Written consent was obtained from all participants at the start of each focus group. The interviews lasted between 56 and 84 min and were recorded using a university-owned audio recording-device.

A semi-structured interview guide with four open-ended questions was prepared in advance. The questions were designed to meet the purpose of the study and explore the usefulness and usability of NANTS-no in clinical practice. The interview started with the moderator asking a general question to start the conversation: “Can you tell me a bit about your experiences using NANTS-no as an assessment instrument in clinical practice?” The follow-up questions were more specific, asking participants to explain how they used NANTS-no, as well as in what ways it might be used to enable critical reflection and dialogue in the learning process.

The SNAs in the two student focus groups all knew each other and the discussion flowed easily with participants sharing experiences and disagreeing with one another. This highlighted similarities and differences in their experiences. The two focus groups with mentors and clinical supervisors were more heterogeneous including representatives from several different healthcare trusts. Although some of the participants were unknown to each other, group interaction in the first group was lively with many of the participants eager to share their experiences. Interaction in the second group however was more constrained, with some participants needing to be prompted to contribute to the discussion. It is unclear why this was the case, but it did not noticeably affect the rich description of their experiences. Humour and laughter played a role in all the groups as a means of easing social constraints and underlining shared experiences [26].

### Data analysis

The interviews were transcribed verbatim and then analyzed using Graneheim and Lundman’s qualitative content analysis [27]. Content analysis is a systematic method for analyzing qualitative data that highlights similarities within and differences between the data. It enables the analysis of both descriptive (manifest) data and interpretative (latent) data that results in categories and/or themes [27].

An inductive approach, which involved immersing oneself in the data was used to search for patterns in the texts and involved a series of steps. First, the transcribed texts were read through several times to gain an initial overview over the data. Any interesting quotes were marked, and notes/comments were made in the margin. The first author also made a flow diagram describing her current understanding of the texts [35]. The next step involved de-contextualizing the data by extracting quotes from the transcribed texts, so-called meaning units, condensing them without altering their meaning, and then assigning them codes [36]. Similar codes were then grouped together into eighteen sub-categories and six categories that represent the manifest content of the interviews. This process was not linear but involved discussion between the authors and movement back and forth between the different parts and the text as a whole. An example of the analysis process is presented in Table 1.

**Table 1:** Illustration of the analysis process from meaning unit to sub-category

Meaning unit	Condensed meaning unit	Code	Sub-category
I realized that this covers much of what we do all the time, what we have done as a nurse anaesthetist the past 30 years, that you always have done…it is just putting it into words	It covers much of what we do as a nurse anaesthetist all the time and have always done. It has just put it into words	Putting into words what a nurse anaesthetist does	Providing a vocabulary for tacit skills
It’s about being able to take over the role that is the function of a nurse anaesthetist… when the mentor is there too. Daring to take that responsibility… that’s asking a lot of students, it’s asking a lot	It’s about taking over the role and function of a nurse anaesthetist while the mentor is still there. Daring to take that responsibility, it’s asking a lot of students	Daring to take over the role and responsibility	Changing behaviour
Since the examples and numbers make it more concrete and measurable, it makes it easier than just the mentor’s gut feeling or view of what a student should be like	Measurable concrete examples and numbers make it easier than a mentor’s gut feeling or view of what a student should be like	Measurable evaluation, not the mentor’s gut feeling	Objectivity
I think it’s a more of a problem for my mentor to give me a low score than for me to give myself a low score	more problematic for mentor to give a low score than for me	Easier for student to give a low score	Scoring barriers

Next followed a process of reflection and discussion on the underlying meaning in the categories to abstract and interpret latent content in the data and go beyond the participants’ actual words. This is often a balancing act with regard to the level of abstraction and degree of interpretation [27, 37]. The categories separated themselves into three specific areas; the way in which use of NANTS-no heighted awareness relating to non-technical skills and professional expertise, the way in which it directly contributed to the mentoring and learning process, and implementation and acceptance of the instrument in clinical practice. Three themes with a relatively low level of abstraction and degree of interpretation were then formulated to describe these areas: Promotion of excellent non-technical skills, Promotion of cooperative learning and Promotion of organizational acceptance. A main theme Forging a path towards clinical excellence*,* with a higher level of abstraction was formulated to encompass the themes and can be interpreted as the latent content or common thread running through the texts [36].

### Ethical considerations

Approval from the Norwegian Centre for Research Data (project no. 854411) was granted on 7.12.2018 and was sufficient for this study [38, 39]. The participants were first informed in writing. At the start of each focus group, the concepts of informed consent, voluntary participation and the right to withdraw without penalty were also carefully explained, in accordance with the Declaration of Helsinki [40] and national guidelines [39]. After time for consideration, informed consent was obtained from all the participants. Since the first author was in a position of authority as program coordinator of the master’s program in nurse anaesthesia, knew all the participants and contacted them directly, it was important to ensure they did not feel coerced [31, 41]. Her role as a researcher in this context was therefore carefully explained, and one of the co-authors assisted at each interview to strengthen credibility. Participants were also informed about requirements regarding confidentiality, data anonymity and secure handling of data.

## Results

**Fig. 2:**
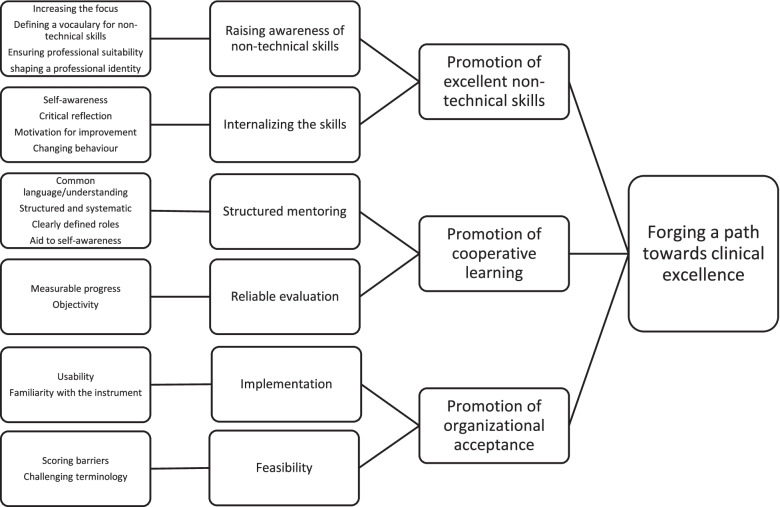
Summary of the analysis of experiences with using NANTS-no (main theme, themes, categories and sub-categories)

NANTS-no was experienced by the participants as a means of promoting excellent non-technical skills and cooperative learning in nurse anaesthesia education. There was however a need for promoting organizational acceptance of the instrument in the working environment. The findings were interpreted as NANTS-no helping to Forge a path towards clinical excellence. A summary of the categories and themes is presented in Fig. 2, and the results are presented for each of the three themes in the text that follows.

In order to demonstrate differences and nuances in the way in which the SNAs, mentors and clinical supervisors experienced using the instrument, quotations are attributed to the relevant participant instead of to the focus group using abbreviations and numbers, for example, student nurse anaesthetist (SNA1), mentor (M6) and clinical supervisor (CS2).

### Promotion of excellent non-technical skills

The participants described using NANTS-no as promoting excellent non-technical skills in student nurse anaesthetists, by raising awareness of the importance of these skills in anaesthesia and helping the students to internalize the skills.

Using NANTS-no was seen as raising awareness by shifting the focus in nurse anaesthesia education to include non-technical skills and providing a vocabulary for these tacit skills. In this way NANTS-no highlighted the skills that were expected of a nurse anaesthetist in their daily work:

“I realized that this covers much of what we do all the time, what you have done as a nurse anaesthetist for the past 30 years, what you have always done…it is just putting it into words.” (M5)

Non-technical skills were experienced as being a part of their professional identity and role as a nurse anaesthetist. However, since non-technical skills could also be regarded as something personal, being judged on behaviour could sometimes be an uncomfortable experience. The mentors and clinical supervisors regarded NANTS-no as a means of ensuring professional suitability and that future colleagues had the right skill-set. One mentor commented that there was no room for poor nurse anaesthetists in such a responsible job, while becoming the same kind of nurse anaesthetist as their mentor was not necessarily seen as a goal by the SNAs. By focusing on non-technical skills, NANTS-no was seen as shaping professional behaviour, and providing a standard of excellence for which to strive throughout their career.

The participants regarded the process of SNAs internalizing these skills as one involving self-awareness and critical reflection, which could then motivate towards changes in behaviour. The first step was for SNAs to become aware of their own strengths and weakness. According to the mentors, the level of self-awareness in SNAs varied, and some took longer than others to gain sufficient insight. The SNAs described using NANTS-no to reflect critically on their clinical progress and align their own view with their mentor’s observations.

“It’s about knowing yourself. Whether your behaviour is on target or way off the mark. Whether you see yourself as others see you. That is not a given, you know” (SNA2).

NANTS-no was experienced as aiding self-awareness by opening the way for a process of dialogue and reflection which could act as a catalyst for enabling change in the SNAs. Many SNAs felt that being rated with a low score and given tangible examples of what needed improving was motivational for both personal and professional development. However, not all the SNAs agreed on this. It was also important to receive encouragement and positive feedback about what they were proficient at.

Changing behaviour was regarded by some mentors as potentially challenging, as it required motivation and willingness to make the necessary effort. A positive aspect of NANTS-no was the way it aided the mentors in making students aware of how they could make changes without it being regarded as criticism of their personality. This was because negative feedback was based on the instrument, not the mentor’s opinion.

“I really wish we had an instrument like this when I was doing my training. I was a quiet, timid kind of person who didn’t dare do much, and my student is just the same. We looked at the team-working category [in NANTS-no], which states you have to take on a role and speak loudly and clearly. So, I wasn’t pointing out something negative about her as a person, or her personality. This was something important.” (M1)

The SNAs described various ways in which they actively worked on improving their non-technical skills, such as taking the lead in an acute situation, being more assertive or trying to be a supportive team-member. However, at the start of their training they regarded it as important to concentrate on just a few NANTS-no elements, then gradually increase the number when they felt ready.

### Promotion of cooperative learning

The participants described using NANTS-no as promoting cooperative learning by enabling both a more structured mentoring process and reliable evaluation of SNAs.

The SNAs experienced NANTS-no as providing a common language for non-technical skills, which made it easier for mentors to follow their progress. Furthermore, it was seen as facilitating a common understanding of what was being observed and assessed.

“We do it together, the assessment. Both of us have the same points that we are assessing – what was your assessment, what was mine, how can we help me to learn these things? So, it’s a kind of professional partnership” (SNA12)

By comparing and discussing their assessments, the mentors and SNAs felt they gained a shared understanding of the student’s clinical progress. According to one of the focus groups, this resulted in a more structured and systematic approach to mentoring. Using NANTS-no enabled the mentors to give the students structured feedback and use their time more efficiently, while simultaneously ensuring that they observed and assessed the students’ non-technical skills.

“I have become very aware of these things myself and use NANTS for all it’s worth as a mentor.” (M1)

(sounds of agreement)

“That’s also what I meant when I said I felt like I have more time. There aren’t any more hours in the day, but it makes it easier to use the few available gaps in between.” (M3)

“In a more structured way, perhaps?” (M2)

(sounds of agreement)

“Because you are able to identify faster the areas, you actually want to… reflect over.” (CS1)

By making the mentors more aware of how they worked together with their students, NANTS-no was seen as changing the way in which they mentored their students. Nevertheless, as one SNA pointed out, whether NANTS-no was used as a mentoring aid was dependent on the mentor. Some mentors still preferred to do things the way they had always done them. The SNAs regarded NANTS-no as contributing to a clearer definition of roles in the mentoring process: “I think it’s a good thing that my mentor is my mentor and not my best friend” (SNA12). They differentiated between the mentor’s and clinical supervisor’s role. Since the supervisor only worked with the students occasionally rather than daily like the mentors, they were regarded as potentially having a different overall view of the students’ progress.

In order to understand what was expected of them at a specific level, the SNAs wanted constructive and specific feedback. By providing tangible examples to explain their assessment, the mentors described using NANTS-no to illustrate where the students demonstrated proficiency as well as to point out what needed improving.

“We sat together, me, my mentor and the clinical supervisor, and went through NANTS. They gave me specific feedback about what they thought I needed to work on. So, in that way I think you learn more than when I took my nursing degree, the feedback then could be rather diffuse and general.” (SNA5)

This kind of structured feedback was regarded as valuable by the SNAs in aiding them to work systematically on improving their non-technical skills. Although the mentors considered NANTS-no made it easier to give negative feedback, the SNAs felt it was difficult for a mentor to be honest when a student had very poor skills.

Although assessing behaviour was regarded as new and unfamiliar by the participants, NANTS-no contributed to a reliable evaluation of the SNAs skills by demonstrating a measurable progress and increasing the objectivity of the evaluation. Regular use of the NANTS-no rating scale made the SNAs’ progress more visible to both the students and their mentors, although some mentors felt that the actual score was not as important as the direction in which it pointed.

Both the SNAs and mentors discussed whether it would be less confusing to use NANTS-no to assess a student’s non-technical skills at the end of each training period in relation to an expected level, rather than as a scale for the whole training. However, using the same scale to measure the SNAs’ progress throughout their training was regarded as one of the most valuable aspects of the instrument.

“… look we agreed that the scale should maybe be organized to score each period, but if one looks at it as an evaluation instrument for the whole training, it’s an advantage to have one scale for the whole thing” (SNA7)

“Yes. I don’t think there is any point having one score for … one dividing it up to rate each period, because you won’t get an overall view then” (SNA11)

The SNAs experienced being evaluated with NANTS-no as less subjective than other clinical evaluations because they were all evaluated on the same objective criteria in a systematic way. This ensured that an evaluation measured their progress more fairly, and was not based on the individual mentor’s hang-ups, gut feelings, or on personal chemistry. However, some SNAs felt that rating skills did not necessarily make the evaluation more objective as a score could always be influenced by the mentor’s or supervisor’s impression of the SNA. The mentors confirmed that they found it difficult to be completely objective when they worked with the student daily, although using NANTS-no encouraged them to reflect over their own behaviour in a more objective way. Nonetheless, they regarded NANTS-no as particularly useful for exemplifying why an SNA failed to pass his or her clinical training.

### Promotion of organizational acceptance

The participants described promoting acceptance of NANTS-no as an ongoing process that encompassed the practicalities and feasibility of implementing the instrument in the anaesthesia departments.

Implementation was regarded as dependent on the instrument’s perceived usability and nurse anaesthetists’ familiarity with it. NANTS-no’s schematic format was described as well organized, giving a complete overview of a nurse anaesthetist’s non-technical skills. Although the SNAs described NANTS-no as compact and requiring little effort to use, many of the mentors found it overwhelming at first. The full-version contained a large amount of text, but this was regarded as necessary to use it optimally. Various participants commented on overlap in some of the NANTS-no elements and categories that hindered familiarization, although the examples of good and poor behaviour helped to clarify the meaning of the elements. However, the participants were generally positive to using NANTS-no.

A major impediment to implementation was seen as lack of familiarity with the instrument, both on an individual and departmental level. Some participants commented that it was only used on “high days and holidays”, and it was like starting afresh each time:

“I would really like to use it a bit more, so I’ve got it under my skin, because I have to go back and look at the elements to see how to score them. I don’t always remember them well enough, so it’s a bit difficult to use it «bedside» without having the instrument with you… but it’s probably a case of practice. If you use it enough, then it will sit properly” (CS4)

Participants experienced that NANTS-no worked well once they were familiar with the instrument, and using it regularly increased their proficiency. The mentors felt that the SNAs had a higher degree of familiarity, and there was a need for better training in the use of NANTS-no. It was also problematic that the instrument was not properly implemented in the anaesthetic department. Gaining acceptance for NANTS-no was described as a maturation process that would take time.

The feasibility of implementing NANTS-no was challenged by scoring barriers and the instrument’s terminology. Rating behaviour and interpersonal skills was regarded as strange and unfamiliar. The mentors regarded it as challenging since providing anaesthesia is a complex process, and an SNA might demonstrate good situational awareness, then miss a small detail that pulled their score down. Using the rating scale to assess whether a student should pass at the end of their training, however, was seen as meaningful. The mentors also expressed concerns about making false judgements and setting too low a score, particularly during the first period of training. It was therefore often easiest to select a score in the middle of the scale.

Although SNAs found using the rating scale became easier over time, it was often difficult to give themselves a high score.

“We find it a bit challenging to put a number on ourselves. At least in my opinion, it’s not always so easy. One is maybe a bit too cautious or too daring rating some of the elements.” (SNA4)

“It has something to do with the Norwegian spirit of egalitarianism, if you like” (SNA1)

Some mentors agreed that the SNAs rated themselves lower than their mentors. However, one clinical supervisor considered that the SNAs understood the rating scale well and were realistic in their self-assessments. She found it surprising that assessments made by three different people often ended up being very similar.

A further challenge for the mentors was using NANTS-no to compare students to a qualified nurse anaesthetist, particularly during their first clinical training period when they could only expect low scores. They accepted however that the SNAs understood the system and found it less problematic. In addition, the terminology in the rating scale was seen as problematic, as the use of «N—not observed» was unclear while some of the rating descriptors at the lower end of the scale such as «poor» or «marginal» were regarded as both harsh and demotivating. However, this was not mentioned as a problem by the SNAs.

## Discussion

The findings in this study provide the first insights into how the structured assessment instrument NANTS-no is experienced, and its usability and usefulness in clinical practice. On a higher level, the study illuminates the way in which using NANTS-no appears to trigger processes and reflections that contribute to the professionalization of both the nurse anaesthetist role and the learning/mentoring process. Although the instrument has not been completely accepted in the challenging environment in which nurse anaesthetists work, there appears to be a high level of commitment to using NANTS-no. These insights may have a transferability value for other healthcare professions interested in integrating a systematic development and assessment of non-technical skills in clinical practice.

Using NANTS-no was described as raising awareness of the importance of non-technical skills. It also provided a tangible and more objective standard of excellence by which SNAs could measure their skills and mentors could determine whether they met expected criteria. The development of non-technical skills is closely interwoven with ideas of professionalism and clinical excellence, which has been an aspirational goal in anaesthesia for the past decade [20, 42]. Aspects of professionalism such as ensuring a high level of knowledge, skills and professional values when delivering patient-centred care, were regarded as prerequisites by the focus groups [5, 9]. Non-technical skills combine these various elements of professional practice into “a coherent performance” [4, 42], ensuring excellence and patient safety.

In the first theme, using NANTS-no to develop excellent non-technical skills was seen as the measure for distinguishing a good nurse anaesthetist from an excellent one. Although the mentors were regarded in general terms as role models, not all the SNAs wanted to model their behaviour on their mentor, as having excellent non-technical skills was not dependent on years of experience. Professionalism includes an active commitment to self-appraisal and continuous professional development [9]. Thus, personal and organizational factors played a significant role, along with the desire to continually improve one’s professional practice [42, 43]. Using NANTS-no also aided the participants in differentiating between the nurse anaesthetist’s role as a professional and private person. Behavioural skills are often regarded as closely associated with an individual’s personality [3], therefore being judged on behaviour could be uncomfortable. However, the participants accepted that the purpose of NANTS-no was changing behaviour and improving skills essential to providing safe anaesthesia care, rather than modifying personality [3].

An important aspect of the mentoring role was seen as determining the SNAs’ professional suitability and aiding them in developing a professional identity as nurse anaesthetists. Therefore, having an instrument that could be used as a means of determining the level of non-technical skills and thus professional suitability was considered a crucial aspect, and one that is relevant in many other healthcare professions. The nurse anaesthetist’s professional identity is described as both mechanistic and supportive, with the nurse anaesthetist protecting and preserving the patient’s integrity and autonomy while monitoring and optimizing physiological functions in a highly technical environment as she “holds the patient’s life in her hands” [44–46]. The complexity of the role and level of responsibility require highly developed non-technical skills such as situation awareness, decision-making, communication and teamwork [22].

Promoting excellent non-technical skills also involved the SNAs internalizing these skills, a process where the behaviour and attitudes of mentors and supervisors were incorporated through learning or assimilation and facilitated change. In the past, non-technical skills have often been addressed in an unstructured manner, lacking a taxonomy that articulated and assessed them systematically [19, 21, 22]. By providing a vocabulary for the tacit qualities of professional expertise, NANTS-no enabled discussion of what the nurse anaesthetist role actually comprises, promoting both excellence and patient safety [42]. A study using a structured assessment instrument for scrub nurses (SPLINTS-no) described heightened awareness as a result of providing a vocabulary for non-technical skills [24]. Articulating their non-technical skills raised the SNAs’ awareness of their own strengths and weaknesses, providing them with the means and motivation to change their behaviour and address any problems. However, internalization of non-technical skills depended on self-awareness, and this was seen as varying. Particularly, when a SNA had difficulties developing clinical skills, a lack of self-awareness and inability to use NANTS-no as intended, was seen by the mentors as contributing factors.

In the second theme, the promotion of cooperative learning was seen as encouraging reflection and critical awareness and depends on a culture of mutual trust and respect, where there is shared responsibility for both the process and the outcome [15, 18]. By enabling a clear definition of roles in the mentoring/learning process, using NANTS-no enabled more objective evaluations as personal chemistry between the student and mentor influenced the outcome to a lesser degree. Furthermore, NANTS-no provided objective criteria for discussion which encouraged cooperation and critical reflection through dialogue. Using the instrument to discuss and articulate their strengths and weaknesses with their mentor and supervisor, heightened the SNAs self-awareness while motivating them to behavioural change [18, 20]. This kind of cooperative learning where the SNA is treated as an equal and a colleague, stimulates professional growth [13].

Using NANTS-no also enhanced the mentoring process by demonstrating a measurable progress and enabling the mentors to give structured and constructive feedback with tangible examples of the SNAs proficiency and what needed to be improved. Addressing behavioural skills and elucidating lack of proficiency can be challenging. However, since feedback was based on the instrument rather than criticism of personal characteristics, NANTS-no was seen as contributing to a more objective and professional form of clinical supervision [24].

Although mentoring students and ensuring sufficient time for reflection was regarded as high priority, the operating room was described as a challenging work environment with production pressure and patient safety conflicts [12]. NANTS-no’s systematic structure was seen as supporting the mentors in their role and facilitating a more effective use of the time available for reflection and dialogue. Interestingly, using NANTS-no also made the mentors more aware of their own professional behaviour, so that it changed the way some of them supervised their students. An increased awareness of the importance of non-technical skills as well as developing their professional role as a nurse anaesthetist and mentor is in line with the IFNA standards [5, 9]. Mentors using SPLINTS-no when supervising student scrub nurses also experienced increased levels of confidence in their role [24].

Instruments such as NANTS-no can promote cooperative learning and develop expert skills by providing a structure for critical reflection, giving formative feedback, and assessing professional behaviour [11, 19, 42]. As one participant stated, using NANTS-no strengthens the professional partnership between student and mentor. However, the pursuit of professionalism in clinical supervision necessitates a commitment to promoting it at all levels in nurse anaesthesia education [20]. It requires among other things a teaching/learning process that encourages active learning, self-reflection and professional growth [20, 43]. Clinical supervision also needs to be a prioritized role supported by formal training programs that facilitate excellent teaching by expert role models, rather than ad-hoc solutions [12, 13, 20].

In the third theme, use of NANTS-no in clinical practice was to a certain extent impeded by the instrument not being fully accepted in the anaesthetic departments. Although generally positive, many nurse anaesthetists were still unfamiliar with NANTS-no, which meant that SNAs were not supervised in the same way if their mentors were not present. A lack of familiarity with non-technical skills and challenges introducing similar structured assessment instruments has been seen in other healthcare professions [24, 47]. In addition, the terminology and use of the rating scale in NANTS-no were seen as negative factors, particularly as rating behavioural skills was unfamiliar. While the mentors expressed concerns that they might rate a student’s skills too low and potentially affect their educational progress, the students were concerned that they might overrate their own skills. This finding was in line with the previous study where the students significantly underrated their non-technical skills [11].

Organizational factors play a major role in ensuring further implementation of this instrument [46]. There is a need for closer cooperation between educational and healthcare institutions to ensure a better understanding of the importance of non-technical skills and promote acceptance and implementation of NANTS-no. To aid this process, educational institutions need to provide sufficient training in observation and assessment of non-technical skills and the use of NANTS-no for clinical staff [30, 47]. Similarly, the anaesthetic departments need to ensure sufficient time for reflection, dialogue, and formative feedback to promote use of NANTS-no and aid SNAs’ professional development.

### Methodological considerations

Trustworthiness in qualitative empirical research is described as encompassing the concepts of credibility, dependability, confirmability and transferability [48]. It should be apparent in all aspects of the research process to ensure the study’s integrity [34]. The first author’s role in the master’s program in nurse anaesthesia at the University and involvement in clinical supervision, in addition to being partially responsible for adapting NANTS-no for use in nurse anaesthesia education in Norway [22] and implementing it at the University, has an impact on the study’s trustworthiness. Her pre-understanding of the context in which the students learn and of the instrument itself, strengthens the study’s credibility by enabling the recruitment of heterogeneous focus groups of relevant individuals with varying experience in using the instrument. This ensured rich variations in the data, which is an important aspect in content analysis [34]. However, the fact that the focus groups were all connected to the same university where the instrument was first adapted and tested may be considered a limitation.

The credibility and dependability of the study may be threatened owing to the first author’s role as a figure of authority and her involvement in recruiting the participants, collecting and analyzing the data [41]. Attempts were made to democratize power relations by informing the participants about the first author’s role as a researcher in this context to avoid feelings of obligation or coercion [41]. In addition, the first author’s role in adapting the instrument may have acted as a bias when interviewing the focus groups and analyzing the data. Attempts were made to counterbalance the first author’s pre-understandings and any potential bias, by the co-authors assisting in conducting the interviews and carrying out open discussions at all levels while analyzing the data [27]. Since the remaining authors are not in any way involved in the clinical side of the master’s program in nurse anaesthesia, this gave them a more open and questioning approach to abstracting and interpreting the data [34].

A possible limitation was the interviews being transcribed by someone outside the research team rather than the researchers. Although this person is regularly employed by the University in this capacity, it may have affected the authors’ analysis and interpretation of the data. However, the authors attempted to immerse themselves in the data by reading the transcripts through while simultaneously listening to the audio files to ensure no valuable data was lost, and to form an impression of the interaction between the participants. Reporting group dynamics in the focus groups, differences of opinions, and how opinions were modified through discussion and consensus achieved, strengthens the study’s dependability [28, 29].

One aspect of strengthening credibility is reporting the way in which data is analyzed and whether the meaning units, categories and themes provided an answer to the study’s aim. Another is striving to prevent incongruence between the degree of interpretation and level of abstraction occurring during data analysis, which could threaten creditability [36]. Being aware of pre-understandings and encouraging a reflexive approach to data collection and analysis strengthens confirmability and the overall trustworthiness of the study. An important aspect of confirmability is that the researchers have taken care to ensure the participants’ voices are heard by using representative quotes to illustrate the findings in the study. However, a possible threat to trustworthiness is that the participants were not invited to read the results and confirm that they recognized the findings presented in the study. In order to determine the transferability of the study a thorough description of how participants were selected, data collected, and the analysis process has been provided [27].

## Conclusion

This study has provided new insights into how the use of NANTS-no is experienced in clinical practice and highlights challenges with using the instrument in nurse anaesthesia education. By increasing awareness of the importance of excellent non-technical skills, using NANTS-no appears to enhance professionalism in the nurse anaesthetist role and promote the ideal of clinical excellence in nurse anaesthesia. It also appears to promote a professional partnership in the mentoring/learning process by clearly defining roles, providing objective criteria for measuring progress, and encouraging cooperation, critical reflection, and dialogue. Heightened self-awareness can lead to change in behaviour and professional development. In this way NANTS-no appears to be useful not only for assessing non-technical skills in nurse anaesthesia education, but also as a means of learning these skills. This study’s findings may therefore be relevant for training other healthcare professionals in non-technical skills. Despite a generally positive attitude towards NANTS-no, ensuring regular use remains challenging, and there is a need to look at improving the terminology and use of the rating scale to promote further acceptance.

## Data Availability

The datasets generated and analyzed during the current study are not publicly available due to reasons regarding confidentiality and anonymity but are available from the corresponding author on reasonable request.

## Abbreviations

ANTS: Anaesthetists’ Non-Technical Skills
ASA: American Society of Anesthesiologists
IFNA: International Federation of Nurse Anesthetists
NANTS-no: Nurse Anaesthetists’ Non-Technical Skills-Norway
SNA: Student nurse anaesthetist
SPLINTS-no: Scrub Practitioners’ List of Intraoperative Non-Technical Skills-Norway
